# The relationship between physical activity and depression among community-dwelling adults in Wuhan, China

**DOI:** 10.3389/fpsyt.2023.1179417

**Published:** 2023-04-25

**Authors:** Kai-Ge Wu, Si-Jing Chen, Ya-Ni Hu, Shu-Fang Mei, Wen-Cai Chen, Xian-E Huang, Zai-Feng Xu, Ming-Chao Li, Bao-Liang Zhong, Xiu-Jun Liu

**Affiliations:** ^1^Institute of Education, China University of Geosciences (Wuhan), Wuhan, China; ^2^Department of Psychiatry, Wuhan Mental Health Center, Wuhan, China; ^3^Department of Psychiatry, Affiliated Wuhan Mental Health Center, Tongji Medical College of Huazhong University of Science and Technology, Wuhan, China; ^4^Research Center for Psychological and Health Sciences, China University of Geosciences (Wuhan), Wuhan, China

**Keywords:** depression, physical activity, mental health, China, female

## Abstract

**Background:**

While the association between physical activity (PA) and depression has been established, there is limited research on the effect of PA on the risk of depression among Chinese individuals. Thus, this study aimed to investigate the relationship between PA and depression among Chinese individuals.

**Methods:**

We used a stratified random sampling approach to recruit participants from five urban districts in Wuhan, China. A total of 5,583 permanent residents aged 18 years or older completed questionnaires, which included the International Physical Activity Questionnaire Short Form (IPAQ-SF) to measure PA, and the 9-item Patient Health Questionnaire (PHQ-9) to evaluate depressive symptoms. To control for potential confounders, multiple logistic regression was employed to assess the association of PA with depression.

**Results:**

The depression group had significantly lower weekly PA levels, measured in metabolic equivalent of task-minutes per week (MET-min/w), compared to the non-depression group [1,770 (693–4,200) MET-min/w vs. 2,772 (1,324–4,893) MET-min/w, *p* < 0.001]. In the fully adjusted model, the moderate and high PA level groups had lower odds ratios (ORs) for depressive symptoms compared to the low PA level group [OR (95% confidence interval (CI)) = 0.670 (0.523–0.858), 0.618 (0.484–0.790), respectively]. Among males, moderate and high levels of PA were associated with lower risk of depression compared to low PA levels [OR (95% CI) = 0.417 (0.268–0.649), 0.381 (0.244–0.593), respectively]. However, this association was not observed in females [OR (95% CI) = 0.827 (0.610–1.121), 0.782 (0.579–1.056), respectively]. The study found a significant interaction between PA levels and gender in relation to depression (*P* for interaction = 0.019).

**Conclusion:**

The findings suggest a negative association between PA and risk of depressive symptoms, indicating that moderate to high levels of PA may serve as a protective factor against depressive symptoms.

## 1. Introduction

Depression is a leading cause of disability worldwide and has a profound impact on the mental health of the general population (1–4). In recent years, several studies have indicated a significant rise in the prevalence of depression (5–7). A 2020 meta-analysis, which included 12 studies, reported a pooled prevalence of 25% for depressive symptoms (8). To develop effective treatments and preventive measures for depression, it is crucial to investigate the patterns of depression and their associated factors.

Research findings indicate that there is a connection between physical activity (PA) and depression, and that PA may serve as a protective factor against depression (9, 10). These effects are thought to be mediated by several mechanisms, including changes in brain structure and function, social interaction and self-efficacy, and improvements in sleep quality (11). Although the most effective level of PA for reducing the risk of depression is still a topic of debate, PA is generally considered beneficial for depression. Some studies suggest that moderate and vigorous PA may both be effective in preventing depression, while other studies raise concerns that high levels of PA may not provide any additional benefits (12–14).

Previous research that examined the link between PA and depression has mostly focused on special subpopulations, such as older adults, teenagers, women, or college students (10, 13–17). However, there is limited evidence from epidemiological studies conducted on large sample populations, particularly among the general adult population in Chinese communities.

The objective of this study was to examine the relationship between PA and depression among a sample of adult residents in China. Considering the differences in prevalence of depression and mediating mechanisms between the genders (18), we aimed to investigate potential gender differences in this association.

## 2. Materials and methods

### 2.1. Study population

Between October and December, 2021, we conducted a cross-sectional household survey of permanent residents aged 18 years and older in Wuhan, Hubei Province, China. Wuhan is a large city with a population of 11.21 million, which is fairly representative of the central region of China. By using a stratified random sampling approach, two central districts and three suburban districts in Wuhan were randomly selected in the first stage of the sampling. From each of the selected districts, we chose two streets, and subsequently, we randomly selected three communities or villages from each street. Within each community or village, we randomly selected 180 households. Finally, we randomly selected one adult participant from each selected household, provided that they had resided in the selected community or village for at least six consecutive months.

Sample size was calculated using the formula N = *deff*·*μ*^2^*p* (1 − *p*)/*d*^2^. A 95% confidence level (two-tailed) was used, corresponding to *u* = 1.96. Based on the research of Lu et al., the prevalence of depression among Chinese was 3.4% (19). The design efficiency (*deff*) was set at 2. The relative error (*r*) was 20%, and *d* was calculated as 0.68 (20% × 3.4%). Using these values, the sample size was determined to be 5,457.

In order to ensure that residents were aware of the procedure and purpose of the study, informed consent was obtained before the survey. The study was approved by the Ethics Committee of Wuhan Mental Health Centre.

### 2.2. Measurements

Trained investigators conducted face-to-face interviews with residents and completed questionnaires on-site. The survey utilized a self-developed online platform to collect data. The questionnaires included information on PA, depressive symptoms, and socio-demographic characteristics.

PA was measured using the International Physical Activity Questionnaire Short Form (IPAQ-SF)—Chinese version in the present study (20). The IPAQ-SF was considered a reliable instrument to assess the total amount of PA obtained in common people aged 15–65 years (21). It is a 7-item self-report scale that calculates daily metabolic equivalents (METs) for the past 7 days. The PA level was classified into three groups (high, moderate, and low) based on the IPAQ-SF scoring protocol (22). IPAQ-SF has been used extensively and its reliability is well-documented (Intraclass Correlation Coefficient (ICC) = 0.79) (20).

The presence and severity of depression were estimated by the Chinese version of the 9-item Patient Health Questionnaire (PHQ-9) (23). This questionnaire has 9 items, and each item was scored on 4 point-scale (0 = Not at all to 3 = Nearly every day) and the total score ranged from 0 to 27. The total score of ≥5, ≥10, ≥15, and ≥ 20 are identified as mild, moderate, moderately severe, and severe depressive symptoms, respectively (23). Thus, this study used a total score of 5 as a cut-off point to generate the binary depression variable. The Cronbach’s alpha of PHQ-9 was 0.863 in this study.

Socio-demographic information collected in the study included gender, age, body mass index (BMI), education level, marital status, monthly family income, smoking status, alcohol drinking status, presence of insomnia, and the number of chronic diseases. The Insomnia Severity Index (ISI) was used to evaluate insomnia status (24), with a cutoff value of 7 and a total score range of 0 to 28 points (25). The internal consistency of ISI was 0.929 for the current study. The number of chronic diseases was determined by a multiple-choice question inquiring whether respondents were currently suffering from any of the 12 chronic diseases listed, including hypertension, gastritis, and diabetes, with an additional option for “other.”

### 2.3. Statistical analyses

For descriptive analysis, Student’s *t*-test was used to compare continuous variables, and differences in categorical variables were compared using the chi-square (χ^2^) test. When continuous data was not normally distributed, the nonparametric Mann–Whitney *U*-test was employed. Logistic regression was then used to examine the association between PA and depression, controlling for potential confounding factors in three models. Model 1 adjusted for gender (female or male), age (continuous), and BMI (continuous); model 2 added marital status (unmarried or married), education (primary school and below, middle school, high school or college and above), monthly family income (<3,000 RMB, 3,000–5,999 RMB, 6,000–9,999 RMB or ≥ 10,000 RMB), current smoking status (no or yes), and current alcohol drinking status (no or yes); and model 3 additionally accounted for insomnia (no or yes) and the number of chronic diseases (0, 1 or ≥ 2). Stratified analyses were performed to evaluate the consistency of the association between PA and depression, considering gender (female or male), age (<60 or ≥ 60 years), BMI (<18.5 kg/m^2^, 18.5–23.9 kg/m^2^, 24.0–27.9 kg/m^2^ or ≥ 28.0 kg/m^2^), monthly family income (<3,000 RMB, 3,000–5,999 RMB, 6,000–9,999 RMB or ≥ 10,000 RMB), insomnia (no or yes) and the number of chronic diseases (0, 1 or ≥ 2). The odds ratios for depression of PA levels were calculated across different subgroups, adjusted for gender, age, BMI, marital status, education, monthly family income, current smoking status, and current alcohol drinking status, insomnia and the number of chronic diseases. Likelihood ratio tests were conducted to examine interactions. All data were analyzed using SPSS 25.0 (IBM Corp., Armonk, NY, United States), and statistical significance was set at *p* < 0.05.

## 3. Results

A total of 5,887 residents were enrolled, and 5,583 completed the assessment. In our study, 765 participants (13.7%) exhibited depressive symptoms. The mean value and standard deviation of age and BMI were 55.66 ± 15.11 years and 23.37 ± 3.33 kg/m^2^, respectively.

The sociodemographic characteristics were compared between individuals with and without depression (Table 1). Compared with non-depression participants, individuals with depression had higher odds of being younger, female, unmarried, and not currently smoking nor drinking alcohol, while with lower levels of BMI, education, and monthly family income. Moreover, individuals with depression had a higher prevalence of chronic diseases, as well as a greater incidence of insomnia. The group reporting depressive symptoms had a significantly lower median (IQR) of PA at 1,770 (693–4,200) MET-min/w compared to the group without depressive symptoms, which had a median (IQR) of 2,772 (1,324–4,893) MET-min/w (*p* < 0.001).

**Table 1:** Demographic characteristics according to depression status.

Parameters	Non-depression (*n* = 4,818)	Depression (*n* = 765)	χ^2^/t/*Z*	*p-*value
Gender, *n*(%)			13.732	<0.001
Female	3,148 (85.1)	552 (14.9)		
Male	1,670 (88.7)	213 (11.3)		
Age (years)	56.1 ± 14.9	53.1 ± 16.4	4.743	<0.001
BMI (kg/m^2^)	23.4 ± 3.3	23.0 ± 3.5	2.963	0.003
Education, *n* (%)			84.945	<0.001
Primary school and below	1,439 (84.4)	265 (15.6)		
Middle school	1,480 (90.6)	153 (9.4)		
High school	1,034 (89.4)	122 (10.6)		
College and above	865 (79.4)	225 (20.6)		
Marital status, *n* (%)			30.738	<0.001
Married	4,139 (87.4)	598 (12.6)		
Unmarried and others	679 (80.3)	167 (19.7)		
Monthly family income, n (%)			19.339	<0.001
<3,000 RMB	1,098 (83.6)	216 (16.4)		
3,000–5,999 RMB	1,037 (85.9)	170 (14.1)		
6,000–9,999 RMB	1,446 (89.1)	177 (10.9)		
≥10,000 RMB	1,237 (86.0)	202 (14.0)		
Current smoking status, *n* (%)			10.487	0.001
No	4,023 (85.7)	674 (14.3)		
Yes	795 (89.7)	91 (10.3)		
Current alcohol drinking status, *n* (%)			4.889	0.027
No	4,168 (85.9)	684 (14.1)		
Yes	650 (88.9)	81 (11.1)		
Insomnia, *n* (%)			1172.54	<0.001
No	4,367 (93.2)	319 (6.8)		
Yes	451 (50.3)	446 (19.7)		
Number of chronic diseases, *n* (%)			88.657	<0.001
0	2,693 (89.1)	328 (10.9)		
1	1,260 (87.1)	187 (12.9)		
≥2	622 (76.5)	191 (23.5)		
Physical activity (MET-min/w)	2,772 (1324–4,893)	1770 (693–4,200)	# -6.068	<0.001

^#^Mann–Whitney *U*-test was conducted.

The relationships were examined between PA levels and depression, as shown in Table 2. In the final model, a significant association between depression and PA was observed after adjusting for gender, age, BMI, marital status, education, monthly family income, current smoking status, current alcohol, insomnia and the number of chronic diseases. Participants with moderate or high level of PA had a significant lower risk of depression than those with low PA level in the fully adjusted model [OR (95% CI) = 0.670 (0.523–0.858), 0.618 (0.484–0.790), respectively].

**Table 2:** Association of physical activity levels with depression.

	Physical activity, OR (95% CI)			*P-*value
	Low	Moderate	High	
Crude	1	0.595 (0.482，0.735)	0.493 (0.400，0.607)	<0.001
Model 1	1	0.610 (0.493，0.754)	0.506 (0.409，0.625)	<0.001
Model 2	1	0.657 (0.529，0.815)	0.550 (0.444，0.682)	<0.001
Model 3	1	0.670 (0.523，0.858)	0.618 (0.484，0.790)	<0.001

Model 1: adjusted for gender, age, BMI. Model 2: additionally adjusted for marital status, education, monthly family income, current smoking status, current alcohol drinking status. Model 3: additionally adjusted for insomnia, number of chronic diseases.

Table 3 presented the adjusted ORs for depression of PA levels by subgroups. The PA-depression association was not significantly differed by age, BMI, insomnia and the number of chronic diseases. In male group, lower ORs for depression of moderate and high PA levels were observed [OR (95%) = 0.417 (0.268–0.649), 0.381 (0.244–0.593), respectively], whereas similar result was not found in female group [OR (95%) = 0.827 (0.610–1.121), 0.782 (0.579–1.056), respectively]. In households with a monthly income of less than 3,000 RMB, moderate to high levels of PA decreased the ORs for depression [OR (95% CI) = 0.434 (0.267–0.706), 0.462 (0.294–0.727), respectively]. However, in the 3,000–5,999 RMB group, the protect role was only found for high PA level [OR (95% CI) = 0.678 (0.387–1.188)], and in the group of monthly family income higher than 6,000, the protect effect was not observed for both moderate and high PA levels [6,000–9,999 RMB: OR (95% CI) = 0.689 (0.408–1.162), 0.705 (0.417–1.191), respectively; ≥10,000 RMB: OR (95% CI) =0.960 (0.594–1.551), 0.944 (0.570–1.565), respectively]. In addition, moderate to high levels of PA had a protective effect for individuals with a BMI of 18.5–23.9 kg/m^2^ [OR (95% CI) = 0.637 (0.460–0.882), 0.579 (0.419–0.799), respectively] and without chronic diseases [OR (95% CI) = 0.620 (0.439–0.877), 0.651 (0.460–0.923), respectively].

**Table 3:** Odd ratios for depression of physical activity levels by subgroups.

Subgroups	*N*	Physical activity			*P* for interaction
		Low	Moderate	High	
Gender					0.019
Female	3,700	1	0.827 (0.610, 1.121)	0.782 (0.579，1.056)	
Male	1,883	1	0.417 (0.268, 0.649)	0.381 (0.244，0.593)	
Age					0.434
≤60	3,136	1	0.724 (0.531, 0.988)	0.644 (0.470，0.882)	
>60	2,447	1	0.554 (0.365, 0.840)	0.583 (0.390，0.870)	
BMI					0.249
<18.5 kg/m^2^	288	1	0.595 (0.233, 1.516)	0.917 (0.369, 2.281)	
18.5–23.9 kg/m^2^	3,130	1	0.637 (0.460, 0.882)	0.579 (0.419, 0.799)	
24.0–27.9 kg/m^2^	1,692	1	0.842 (0.515, 1.376)	0.624 (0.382, 1.020)	
≥28.0 kg/m^2^	473	1	0.362 (0.142, 0.924)	0.740 (0.309, 1.769)	
Monthly family income					0.311
<3,000 RMB	1,314	1	0.434 (0.267, 0.706)	0.462 (0.294, 0.727)	
3,000–5,999 RMB	1,207	1	0.678 (0.387, 1.188)	0.539 (0.313, 0.927)	
6,000–9,999 RMB	1,623	1	0.689 (0.408, 1.162)	0.705 (0.417, 1.191)	
≥10,000 RMB	1,439	1	0.960 (0.594, 1.551)	0.944 (0.570, 1.565)	
Insomnia					0.871
No	4,686	1	0.637 (0.463, 0.877)	0.606 (0.442, 0.831)	
Yes	897	1	0.670 (0.452, 0.995)	0.605 (0.411, 0.890)	
Number of chronic diseases					0.768
0	3,021	1	0.620 (0.439, 0.877)	0.651 (0.460, 0.923)	
1	1,447	1	0.710 (0.421, 1.197)	0.688 (0.415, 1.141)	
≥2	813	1	0.696 (0.419, 1.156)	0.546 (0.333, 0.896)	

All models are adjusted for gender, age, BMI, marital status, education, monthly family income, current smoking status, and current alcohol drinking status, insomnia and the number of chronic diseases.

## 4. Discussion

The present study investigated the association between PA and depression among Chinese adults. We observed a significant negative correlation between PA level and odds of depression, although this association was only evident in men but not in women.

Our study found a prevalence rate of 13.7% for depressive symptoms among permanent residents aged 18 years or older in Wuhan, China. Our results are consistent with previous studies, which have also shown that depressive symptoms are more common in females than in males (14.9% vs. 11.3%) (13, 26). This may be attributed to females’ high sensitivity to psychological and physical stress, which may increase their susceptibility to depression, particularly during emergencies (18, 27).

Our study revealed that both moderate and high levels of PA were associated with a lower risk of depressive symptoms. These findings are consistent with the results of previous studies by Chi et al. and Mumba et al., which have shown that moderate to high levels of PA are associated with lower levels of depression compared to lower levels of PA (13, 14). In contrast, other studies have shown that vigorous PA does not have additional protection against depression and that it may increase the risk of depression (12, 28). Our results supported the former view, suggesting that both moderate and high levels of PA had a protective effect against depression, and the risk of depression decreased with increasing levels of PA. Many studies have demonstrated the positive effects of PA on mental health through several neurobiological, psychosocial, and behavioral mechanisms. The neurobiological mechanism hypothesis suggests that PA results in changes in the structure and function of the brain, which enhances mental health (11, 29). The psychosocial mechanism hypothesis proposes that PA provides opportunities for social interaction, self-efficacy, and improved body image, leading to better mental health (30, 31). Lastly, the behavioral mechanism hypothesis posits that engaging in vigorous PA may increase the need for sleep, which promotes energy conservation and body restoration, ultimately contributing to better mental health (32, 33).

According to our findings, PA may not have protective effect on women, which differed from previous studies. Meng et al.’s study of 1,892 elderly women (aged 55–70) found that moderate leisure-time physical activity (LTPA) was associated with reduced depression, whereas vigorous LTPA could increase the risk (28). Similarly, Luo et al.’s study on middle-aged women (aged 42–52) found that adequate PA (at least 150–300 min of moderate-intensity aerobic activity per week) was a protective factor against depressive symptoms (17). The protective effect of PA on women in our study was not significant, probably due to the type of PA performed. Several studies have shown that aerobic and strength exercises are the most effective in alleviating depression (34, 35). However, it is possible that some of the individuals in our study may have reported doing household chores as part of their PA. Previous studies have suggested that increased workload and lack of skill utilization during household chores may be risk factors for depression in women (36), and that women with high household workload have higher rates of mental disorders than women with low household workload (37). Moreover, the age range of women in this study was wider (18–95 years). The effect of PA may vary among women of different age groups, which calls for further research.

Our study suggested that the protective effect of PA against depression may vary across income groups. PA had no significant protective effect against depression in high-income households, but had a significant protective effect in low-income households. PA may be an inexpensive and feasible way to reduce depression in low-income households. Conversely, high-income households may have more options available to alleviate depression, which could reduce the protective effect of PA. As for the role of BMI and number of chronic diseases in the relationship between PA and depression, further research is needed due to the small sample size in some of the subgroups of these variables.

Several limitations of the present study should be acknowledged. Firstly, the cross-sectional design of our study precluded making causal inferences, even though we controlled for relevant covariates. Future research should consider more comprehensive longitudinal studies to better understand the relationships between the variables. Secondly, PA was collected through self-reported instruments, which may be subject to biases resulting from social expectations and memory errors. Thirdly, it should be noted that the PHQ-9 assessment of depression served as a screening tool and does not provide a clinical diagnosis. As a result, future studies may need to incorporate diagnostic interviews to ensure that results are more precise and reliable. Fourthly, the study only involved permanent residents in Wuhan, which may limit the generalizability of our findings to residents of other Chinese provinces due to differences in economy and living habits.

## 5. Conclusion

PA is negatively associated with depression. The risk of depression decreases with the increasing of PA, and both moderate and high levels of PA may play a protective role. Further study is warranted to clarify the sex difference in the effect of PA.

## Data availability statement

The original contributions presented in the study are included in the article/Supplementary material, further inquiries can be directed to the corresponding author.

## Ethics statement

The studies involving human participants were reviewed and approved by the Ethics Committee of Wuhan Mental Health Centre. The patients/participants provided their written informed consent to participate in this study.

## Author contributions

B-LZ, X-JL, S-JC, Y-NH, S-FM, and W-CC designed the research. K-GW, S-JC, Y-NH, S-FM, W-CC, X-EH, Z-FX, and M-CL collected the data. K-GW and S-JC contributed to the data analysis. K-GW wrote the paper. S-JC reviewed the final version of the manuscript and supervised the project. All authors contributed to the article and approved the submitted version.

## Conflict of interest

The authors declare that the research was conducted in the absence of any commercial or financial relationships that could be construed as a potential conflict of interest.

## Publisher’s note

All claims expressed in this article are solely those of the authors and do not necessarily represent those of their affiliated organizations, or those of the publisher, the editors and the reviewers. Any product that may be evaluated in this article, or claim that may be made by its manufacturer, is not guaranteed or endorsed by the publisher.
